# Impact of the number of previous embryo implantation failures on IVF/ICSI-ET pregnancy outcomes in patients younger than 40 years: a retrospective cohort study

**DOI:** 10.3389/fendo.2023.1243402

**Published:** 2023-09-29

**Authors:** Yuan Fang, Fan Jingjing, Cheng Tiantain, Xie Huanhuan, He Qiaohua

**Affiliations:** ^1^ Reproductive Medicine Center, Henan Provincial People's Hospital, Zhengzhou, China; ^2^ People's Hospital of Henan University, Zhengzhou, China; ^3^ People's Hospital of Henan University, People's Hospital of Zhengzhou University, Zhengzhou, China

**Keywords:** IVF/ICSI, implantation rate (IR), Live birth rate (LBR), pregnancy outcome, recurrent implantation failure (RIF)

## Abstract

**Objective:**

The objective of this study was to examine the influence of repeated embryo implantation failures on pregnancy outcomes among patients under 40 years of age undergoing *in vitro* fertilization/intracytoplasmic sperm injection embryo transfer (IVF/ICSI-ET).

**Materials and methods:**

A retrospective analysis was conducted on the clinical data of 13,172 patients who underwent 16,975 IVF/ICSI-ET treatment cycles at Henan Reproductive Hospital between January 1, 2015, and December 31, 2018. Patients were categorized into four groups based on the number of previous embryo implantation failure cycles: Group A=no implantation failure, Group B= 1 implantation failure, Group C=2 implantation failures, Group D=≥3 implantation failures. Baseline characteristics and pregnancy outcomes were compared among the four groups. The impact of the number of previous embryo implantation failures on pregnancy outcomes among IVF/ICSI-ET patients was investigated using univariate and multiple regression analyses.

**Results:**

Univariate logistic regression analysis demonstrated that factors such as the number of previous embryo implantation failures, female age, basal follicle count, endometrial thickness, total number of oocytes retrieved, type of cycle, number of high-quality embryos transferred, and stage of embryo development significantly affected implantation rate, clinical pregnancy rate, early spontaneous abortion rate, and live birth rate (all *P* < 0.05). The duration of infertility and anti-Mullerian hormone (AMH) levels were also found to influence implantation rate, clinical pregnancy rate, and live birth rate (all *P* < 0.05). Upon conducting multivariate logistic regression analysis and adjusting for confounding factors such as age, AMH levels, basal follicle count, endometrial thickness, total number of oocytes obtained, cycle type, number of high-quality embryos transferred, ovarian stimulation protocol, and stage of embryo development, it was revealed that, compared to Group A, Groups B, C, and D exhibited significantly lower implantation and live birth rates, as well as a significantly higher risk of early spontaneous abortion (all *P* < 0.05).

**Conclusions:**

The number of previous embryo implantation failures is an independent factor affecting implantation rate, clinical pregnancy rate, spontaneous abortion rate and live birth rate of patients underwent IVF/ICSI-ET. With the increase of the number of previous embryo implantation failures, the implantation rate, clinical pregnancy rate and live birth rate of patients underwent IVF/ICSI-ET decreased significantly, and the rate of early spontaneous abortion gradually increased.

## Introduction

Infertility has increasingly become a significant global public health and sociological issue that profoundly impacts human development and health (1). The World Health Organization reports that infertility affects approximately 8–14% of women of reproductive age in western developed countries, with the prevalence rising to 25–30% in some developing regions, such as Africa and the Middle East (2). Currently, the infertile population in China exceeds 40 million, with an incidence of 12.5% among women of childbearing age (3). With the rapid advancement of assisted reproductive technology (ART), the implantation rate for a single high-quality blastocyst transfer has improved to as high as 65% (4). The *in vitro* fertilization/intracytoplasmic sperm injection-embryo transfer (IVF/ICSI-ET) technique has helped numerous infertile families achieve a healthy baby. However, approximately 10% of these individuals are unable to attain a clinical pregnancy even after three or more embryo transfers (5). Despite facing considerable emotional and financial strain, most of these patients still desire to continue their IVF/ICSI-ET treatment in hopes of increasing the “take-home-baby” rate.

Numerous factors influence the embryo implantation rate, including embryo chromosomal abnormalities, diminished endometrial receptivity, asynchrony between embryo and endometrium, maternal endocrine disorders, and thrombotic tendencies. The number of previous embryo implantation failures also plays an essential role in the embryo implantation rate (6). As the number of previous embryo implantation failures rises, the financial and psychological burden on patients grows as well. These individuals require clinicians to help predict the success probability of subsequent embryo transfer cycles based on the number of previous embryo implantation failures. However, the impact of the number of previous embryo implantation failures on the pregnancy outcome of the next IVF/ICSI-ET for infertile patients remains a contentious issue (7–11). Consequently, our study aimed to investigate the effect of the number of prior embryo implantation failures on the pregnancy outcome of the subsequent IVF/ICSI-ET treatment for patients aged <40 years.

## Materials and methods

### Patient and cycle characteristics

The present study analyzed the clinical data of 16,975 cycles of IVF/ICSI from 13,172 patients who were recruited from the Center of Henan Reproductive Hospital between January 2015 and December 2018. The inclusion criteria comprised the presence of indications for IVF/ICSI-ET and the absence of contraindications, in addition to an age < 40 years. The exclusion criteria included incomplete data or loss to follow-up, ultrasound-identified abnormal uterine structure, such as single or bicornuate uterine diaphragm, chromosomal abnormalities in either partner, previous pregnancy from IVF/ICSI-ET cycles, and the use of donor eggs or sperm. The IVF/ICSI-ET cycles were categorized into four groups according to the number of previous embryo implantation failures: Group A included 13172 cycles without any implantation failures; Group B included 2989 cycles with one embryo implantation failure; Group C included 658 cycles with two embryo implantation failures; and Group D included 156 cycles with three or more embryo implantation failures. The Ethics Committee of Henan Provincial People’s Hospital approved the study (No. (28) Len Audit (2022).

### Controlled ovarian stimulation protocol

The appropriate protocol for controlled ovulation was selected based on the patient’s age, body mass index (BMI), basal follicle count, basal sex hormone levels, anti-müllerian hormone (AMH) levels, and previous IVF/ICSI-ET protocols. Patients underwent controlled ovulation stimulation (COS) and the dosage of gonadotropin was adjusted according to follicular size and hormonal levels. When the follicular diameter was ≥18 mm in three or more follicles, 4000–10000 IU of human chorionic gonadotropin (hCG) (Lizhu Pharmaceutical Company, China) was administered to induce ovulation. Oocyte retrieval guided by vaginal ultrasound was performed 36–37 h later. Routine luteal support was performed after oocyte retrieval, and one to two available embryos were transferred on either the third or fifth day after oocyte retrieval.

### Preparation protocols for frozen-thawed ET

Patients with regular menstrual cycles and normal ovulation underwent natural cycle preparation of the endometrium. Transvaginal ultrasound was used to monitor follicular development and endometrial condition starting from day 11 of the menstrual cycle. Endometrial transformation occurred on the day of ovulation, and the endometrium was transformed 3 or 5 days before the transfer of cleavage-stage or blastocyst-stage embryos, respectively. For patients with irregular menstruation, hormone replacement therapy was employed using estradiol valerate (Progynova, 1 mg/tablet, Bayer, Germany). Oral administration of 4–8 mg/day was started on day 2–4 of the menstrual cycle. The dose of Progynova was maintained when the endometrial thickness was ≥8 mm, and the duration of administration was ≥11 days, while luteal support was given at the same time. After progesterone was administered, embryos were transferred on the third or fifth day. Endometrial transformation was performed on day 4 or 6 for the transfer of cleavage-stage or blastocyst-stage embryos, respectively.

### Conventional luteal phase support regimen

Luteal support was provided through daily administration of vaginal progesterone gel (Crinone, 90 mg/unit, Merck & Serono) at a dosage of 90 mg, along with a once-daily oral dose of 20 mg dydrogesterone. The estrogen and progesterone doses remained unchanged after embryo transfer until blood β-hCG levels were measured on the 14th day post-embryo transfer. In case of pregnancy (β-hCG > 50 U/L), the original luteal support was maintained and the dosage was gradually reduced until the 10th week of pregnancy.

### Clinical observation indicators

Clinical pregnancy was defined as the presence of an intrauterine gestational sac, confirmed by vaginal ultrasonography 4–6 weeks after embryo transfer. Spontaneous miscarriage was defined as pregnancy termination before 28 weeks of gestation with a fetal weight of less than 1000g. When spontaneous miscarriage occurs before 12 weeks of gestation, it is considered early pregnancy loss. Newborns delivered after 28 weeks of gestation and surviving are considered live births.

Other observed indicators included age, duration of infertility, BMI, type of infertility, cycle type, stage of embryo development, number(No.) of embryos transferred, endometrial thickness, No. of good-quality embryos, AMH levels, antral follicle count (AFC), infertility factors, and ovulation protocol.

### Statistical analysis

Statistical analysis was conducted using Empower Stats software based on R language. Continuous variables were presented as mean ± standard deviation (SD), and categorical variables were presented as N (%). Intergroup comparisons were performed using one-way analysis of variance (ANOVA) and χ^2^ tests for categorical variables. Univariate regression analysis was used to identify potential factors affecting pregnancy outcomes. Furthermore, multivariate Logistic regression model with generalized estimating equation (GEE) was chosen to conduct univariate and multivariate analysis of the association of effect of the number of previous embryo implantation failures on pregnancy outcomes to account for the correlation between cycles from the same patient and to obtain ORs and 95% CIs for the risk of the number of implantation failures associated with pregnancy outcomes, while adjusting for confounding factors. A value of *P <* 0.05 was considered statistically significant.

## Results

A total of 13,172 patients who underwent 16,957 IVF/ICSI cycles were recruited and categorized into four groups based on the number of previous embryo implantation failures. The patient demographics and characteristics are presented in **Table 1**. The four groups exhibited significant differences in terms of age, duration of infertility, AMH levels, AFC, endometrial thickness, number of eggs retrieved, stage of embryo development, infertility factors, No. of high-quality embryos transferred, cycle type, and ovarian stimulation protocol (*P* < 0.05). However, no significant differences were observed in BMI, type of infertility, No. of embryos transferred, or No. of quality embryos transferred among the groups (*P* > 0.05).

**Table 1:** Comparison of demographic and clinical characteristics of the four groups of patients.

Item	group A (n = 13172)	group B (n = 2989)	group C (n = 658)	group D (n = 156)	*P-value*
Age(y)	30.25 ± 4.15	31.09 ± 4.29^a^	31.84 ± 4.18^ab^	33.41 ± 4.10 ^abc^	<0.001
BMI (kg/m2)	23.32 ± 4.69)	23.45 ± 3.84	23.22 ± 3.54	23.37 ± 5.29	0.591
Duration of infertility((y))	3.95 ± 2.77	2.88 ± 3.11 ^a^	3.04 ± 3.04 ^a^	3.47 ± 3.09^ab^	<0.001
AMH(ng/mL)	4.48 ± 3.76	3.20 ± 1.77 ^a^	3.04 ± 1.90^a^	2.96 ± 1.92^a^	<0.001
AFC	12.85 ± 6.24	8.52 ± 7.85^a^	8.68 ± 7.37 ^a^	7.92 ± 6.31^a^	<0.001
Endometrial thickness(mm)	10.25 ± 2.64	9.83 ± 2.38^a^	9.69 ± 1.92^a^	9.59 ± 2.09^a^	<0.001
No. of Oocytes retrieved	8.58 ± 3.89	8.22 ± 3.49	4.58 ± 5.11^ab^	5.61 ± 4.94^ab^	<0.001
Types of Infertility					0.078
Primary infertility	4750(53.59%)	697 (51.63%)	109 (45.99%)	25 (53.19%)	
Secondary Infertility	4114(46.41%)	653 (48.37%)	128 (54.01%)	22 (46.81%)	
Cause of infertility					0.014
PCOS	968 (12.02%)	197 (10.35%)	29 (11.37%)	3 (4.84%)	0.117
Tubal factors	4715 (58.54%)	1140 (61.37%)	155 (60.78%)	38 (61.29%)	0.193
Ovulatory dysfunction	909 (11.29%)	212 (11.37%)	27 (10.59%)	7 (11.29%)	0.987
Endometriosis	255 (3.17%)	51 (2.74%)	7 (2.75%)	1 (1.61%)	0.686
Male factors	785(9.75%)	191(10.25%)	32 (12.54%)	10 (16.12%)	0.160
Other factors	375 (4.66%)	63 (3.38%)	3 (1.19%)	1 (1.61%)	0.001
Unexplained infertility	47 (0.57%)	10 (0.54%)	2 (0.78%)	2(3.24%)	0.282
Ovarian stimulation protocol					0.004
long protocol	7756 (60.138%)	1776 (60.801%)	390 (60.278%)	87 (57.237%)	0.877
antagonist protocol	1642 (12.732%)	340 (11.640%)	92 (14.219%)	14 (9.211%)	0.121
mild ovarian stimulation protocol	759 (5.885%)	158 (5.409%)	32 (4.946%)	2 (1.316%)	0.063
PPOS	438 (3.396%)	91 (3.115%)	26 (4.019%)	12 (7.895%)	0.012
Other protocol	2302 (17.849%)	556 (19.035%)	107 (16.538%)	37 (24.342%)	0.061
stage of embryo development					<0.001
Cleavage embryo	10267(77.97%)	1657 (55.49%)	308 (47.17%)	82 (52.90%)	
Blastocyst	2901(22.03%)	1329 (44.51%)	345 (52.83%)	73 (47.10%)	
No. of embryos transferred					0.817
1	3417 (25.94%)	767 (25.69%)	175 (26.80%)	36 (23.23%)	
2	9754 (74.06%)	2219 (74.31%)	478 (73.20%)	119 (76.77%)	
No. of high-quality embryos transferred	1.68 ± 0.51	1.54 ± 0.50	1.51 ± 0.49	1.59 ± 0.50	0.213
No. of high-quality embryos transferred(%)	66.98%(15334/22921)	59.11%(3077/5205)^a^	57.17% (646/1131)^a^	47.31% (130/276)^abc^	p<0.001
Type of cycle					p<0.001
Fresh cycle	4267(50.5%)	417(20.5%)^a^	91(17.9%)^a^	30 (21.3%)^a^	
Frozen cycle	4184(49.5%)	1616(79.5%)^a^	416 (82.1%)^a^	111(78.7%)^a^	

overall comparison between the four groups *P < 0.05; a: compared with group A, *P < 0.05; b: compared with group B, *P < 0.05; c: compared with group C, *P < 0.05.

### Comparison of clinical outcomes among the four groups

The pregnancy outcomes of the four groups are presented in **Table 2**. The implantation rate, clinical pregnancy rate, and live birth rate exhibited significant decreases with an increase in the number of previous embryo implantatfailures (*P<0.001*). Conversely, the rate of early spontaneous abortion increased significantly (*P<0.001*).

**Table 2:** Comparison of clinical pregnancy outcomes among the four groups of patients.

Item	group A	group B	group C	group D	*P -value*
Implantation rate (n%)	11457/22928 (49.97%)	2085/5211 (40.01%)^a^	436/1132 (38.52%))^a^	72/276 (26.09%))a^bc^	<0.001
Pregnancy rate (n%)	8491/13172 (64.46%)	1561/2989 (52.22%)^a^	336/658 (51.06%)^a^	57/157 (36.54%)^abc^	<0.001
Early spontaneous abortion rate (n%)	1152/8491 (13.57%)	265/1561 (16.97%)^a^	74/336 (22.02%)^ab^	16/57 (28.07%)^abc^	<0.001
Live birth rate (n%)	7261/13172 (54.78%)	1275/2989 (42.66%)^a^	258/658 (39.21%)^a^	39/156 (25.00%)^abc^	<0.001

overall comparison between the four groups *P < 0.05; a: compared with group A, *P < 0.05; b: compared with group B, *P < 0.05; c: compared with group C, *P < 0.05.

### Univariate analysis for clinical outcomes

The univariate logistic regression analysis revealed that the number of previous embryo implantation failures, female age, AFC, endometrial thickness, total number of eggs obtained, cycle type, No. of high-quality embryos transferred, and stage of embryo development had an impact on the implantation rate, clinical pregnancy rate, early spontaneous abortion rate, and live birth rate (*P* < 0.05). The factors affecting implantation, clinical pregnancy, and live birth rates were duration of infertility, AMH levels, and AFC (*P* < 0.05). (**Table 3**)

**Table 3:** Univariate analysis of clinical outcomes affecting the number of previous embryo implantation failures.

Item	Implantation rate		Pregnancy rate		Early spontaneous abortion rate		Live birth rate		
	OR (95% CI)	*P -value*	OR (95% CI)	*P -va*lue	OR (95% CI)	*P -value*	OR (95% CI)	*P-value*	
Duration of infertility (y)	0.98 (0.97,1.00)	0.009^*^	0.98 (0.97, 1.00)	0.008*	1.02 (1.00,1.04)	0.120	0.98 (0.97,0.99)	0.002^*^	
AMH (ng/mL)	1.05 (1.03, 1.06)	<0.000*	1.05 (1.03, 1.06)	<0.000*	0.99 (0.97,1.01)	0.301	1.04 (1.03,1.05)	<0.000^*^	
AFC	1.03 (1.03, 1.04)	<0.000*	1.03 (1.03, 1.04)	<0.000^*^	0.99 (0.98,1.00)	0.045	1.03 (1.02,1.03)	<0.000^*^	
Endometrial thickness (mm)	1.07 (1.05, 1.08)	<0.000^*^	1.07 (1.05, 1.08)	<0.000^*^	0.94 (0.92,0.97)	<0.000^*^	1.07 (1.06,1.09)	<0.000^*^	
No. of Oocytes retrieved	1.04 (1.03, 1.05)	<0.000^*^	1.04 (1.03, 1.05)	<0.000^*^	0.95 (0.94,0.97)	<0.000^*^	1.05 (1.04,1.06)	<0.000^*^	
Cause of infertility (n%)									
PCOS	1	1	1	1	1	1	1	1	
Tubal factors	0.79 (0.69, 0.90)	0.000^*^	0.79 (0.69, 0.90)	0.001^*^	0.77 (0.63,0.95)	0.013^*^	0.91 (0.80,1.03)	0.139	
Ovulatory dysfunction	0.77 (0.65, 0.91)	0.002^*^	0.78 (0.66, 0.93)	0.005^*^	0.78 (0.59,1.04)	0.088	0.88 (0.75,1.04)	0.128	
Endometriosis	0.73 (0.57, 0.95)	0.019^*^	0.72 (0.56, 0.94)	0.015^*^	0.80 (0.51,1.25)	0.333	0.86 (0.67,1.10)	0.228	
Male factors	1.25 (1.01, 1.55)	0.043^*^	1.23 (0.99, 1.53)	0.056	0.59 (0.42,0.84)	0.003^*^	1.44 (1.18,1.76)	0.001^*^	
Other factors	0.98 (0.78, 1.24)	0.882	1.02 (0.81, 1.30)	0.845	0.55 (0.36,0.84)	0.006	1.19 (0.95,1.49)	0.122^*^	
Unexplained infertility	1.04 (0.59, 1.84)	0.882	1.03 (0.59, 1.82)	0.914	0.86 (0.35,2.07)	0.730	1.06 (0.62,1.79)	0.836	
Ovarian stimulation protocol									
long protocol	1	1	1	1	1	1	1	1	
antagonist protocol	1.01 (0.92, 1.11)	0.826	1.01 (0.92, 1.11)	0.870	0.95 (0.80,1.13)	0.586	0.64 (2.92,7.38)	<0.000^*^	
mild ovarian stimulation protocol	1.07 (0.93,1.22)	0.358	1.07 (0.93, 1.22)	0.357	0.83 (0.64,1.06)	0.138	0.58 (1.96,6.57)	<0.000^*^	
PPOS	1.07 (0.89, 1.27)	0.458	1.08 (0.91, 1.29)	0.370	1.13 (0.89,1.57)	0.247	0.67 (1.92,1.37)	0.001^*^	
Other protocol	1.02 (0.94, 1.11)	0.708	1.03 (0.95, 1.12)	0.529	0.86 (0.74,1.00)	0.053	0.48 (0.32,0.44)	<0.000^*^	
Type of cycle (n%)									
Fresh cycle	1	1	1	1	1	1	1	1	
Frozen cycle	0.81 (0.75, 0.88)	<0.000^*^	0.81 (0.75, 0.88)	<0.000^*^	1.43 (1.24,1.64)	<0.000^*^	0.77 (0.71,0.83)	<0.000^*^	
No. of high-quality embryos transferred (%)	1.24 (1.41,1.52)	<0.000*	1.23 (1.31,1.65)	<0.000*	0.95 (1.31,1.52)	<0.000*	1.18 (1.15,1.57)	<0.000*	
Stage of embryo development									
Cleavage embryo	1	1	1	1	1	1	1	1	
blastocyst	1.25 (1.16,1.34)	<0.000^*^	1.23 (1.15, 1.32)	<0.000^*^	1.27 (1.13,1.43)	<0.000^*^	1.11 (1.04,1.19)	0.002^*^	

OR: ratio; CI is confidence interval, *P<0.05.

### Multiple logistic regression analysis

Multiple logistic regression was performed to analyze the impact of the number of previous embryo implantation failures on the implantation, clinical pregnancy, early spontaneous abortion, and live birth rates after IVF/ICSI-ET, while adjusting for confounding factors such as age, duration of infertility, AMH levels, AFC, endometrial thickness, total number of oocytes retrieved, infertility factors, embryonic developmental stage, and cycle type. The implantation rate, clinical pregnancy rate, and live birth rate of patients in groups B, C, and D exhibited a gradual decrease compared to group A, while early spontaneous abortion rates showed a significant increase. (**Table 4**)

**Table 4:** Multifactorial logistic regression analysis of the number of previous embryo implantation failures on pregnancy outcome of IVF/ICSI-ET patients.

Item	Unadjusted		Adjusted	
	OR (95% CI)	*P* -value	OR (95% CI)	*P-* value
Implantation rate				
group A	1	1	1	1
group B	0.61 (0.56, 0.66)	<0.001*	0.57 (0.51, 0.63)	<0.001*
group C	0.58 (0.50, 0.68)	<0.001*	0.53 (0.44, 0.64)	<0.001*
group D	0.32 (0.23, 0.45)	<0.001*	0.33 (0.23, 0.46)	<0.001*
Pregnancy rate				
group A	1	1	1	1
group B	0.60 (0.56, 0.65)	<0.001*	0.57 (0.51, 0.63)	<0.001*
group C	0.58 (0.49, 0.67)	<0.001*	0.53 (0.44, 0.63)	<0.001*
group D	0.32 (0.23, 0.44)	<0.0001*	0.32 (0.23, 0.46)	<0.0001*
Early spontaneous abortion rate				
group A	1	1	1	1
group B	1.27 (1.10, 1.46)	0.009*	1.40 (1.16, 1.70)	< 0.001*
group C	1.71 (1.32, 2.22)	<0.001*	1.84 (1.36, 2.48)	<0.001*
group D	2.61 (1.49, 4.58)	0.008*	2.24 (1.24, 4.02)	0.073*
Live birth rate				
group A	1	1	1	1
group B	0.61 (0.57, 0.67)	<0.001*	0.56 (0.50, 0.63)	<0.001*
group C	0.53 (0.45, 0.62)	<0.001*	0.49 (0.41, 0.58)	<0.001*
group D	0.28 (0.19, 0.40)	<0.001*	0.29 (0.20, 0.42)	<0.001*

Group A was used as the reference group. *P < 0.05 after adjusting for confounding factors, including age, duration of infertility, AMH levels, AFC, endometrial thickness, the total number of eggs obtained, infertility factors, embryo development stage, cycle type, ovarian stimulation regimen, and the number of good-quality embryos transferred.

## Discussion

The impact of the number of previous implantation failures on pregnancy outcomes remains a controversial topic that requires further investigation. Cimadomo Danilo et al (12) reported in an observational study that the embryo implantation rate and early spontaneous abortion rate in the first four cycles of IVF/ICSI-ET treatment could not be predicted based on the number of previous embryo implantation failures. However, they found that the live birth rate decreased significantly in patients with a history of ≥3 implantation failures compared to those who underwent their first IVF/ICSI cycle. A retrospective cohort study in Israel reported that the live birth rate per IVF/ICSI-ET cycle decreased significantly with the increasing number of embryo transfer cycles. The study found that the live birth rate was almost zero in patients who underwent the fourth embryo transfer cycle, suggesting that infertile patients who did not achieve a clinical pregnancy with four consecutive embryo transfer cycles should discontinue treatment or consider alternative options such as donor eggs and sperm (7). Conversely, a large multicenter study by Andrew D. A. C. Smith et al (8) showed that the success rate of IVF/ICSI-ET tended to decrease with the number of embryo transfer cycles until the ninth cycle, after which live birth was almost impossible. They support the notion that, although the live birth rate decreased after 3–4 cycles of repeated treatment, there was still a possibility of live birth. Wang et al. (10) found no significant correlation between clinical pregnancy rate and the number of the first three transfer cycles, but observed significantly lower implantation and clinical pregnancy rates from the fourth embryo transfer cycle. The inconsistencies among these studies may be due to differences in study population characteristics, inclusion and exclusion criteria, quality of transferred embryos, level of assisted reproductive technology in each country, and sample size. Our findings demonstrate that the number of previous embryo implantation failures is an independent factor affecting implantation rate, clinical pregnancy rate, spontaneous abortion rate and live birth rate of patients underwent IVF/ICSI-ET. With the increase of the number of previous embryo implantation failures, the implantation rate, clinical pregnancy rate and live birth rate of patients underwent IVF/ICSI-ET decreased significantly, and the rate of early spontaneous abortion increased markedly.

### Effect of the number of previous embryo implantation failures on implantation, clinical pregnancy, and live birth rates in IVF/ICSI-ET patients

Embryo implantation is a crucial step in the success of IVF/ICSI-ET treatment. However, recurrent embryo implantation failure (RIF) is a challenging condition that hinders the improvement of clinical pregnancy rates in these patients. Diagnostic and therapeutic challenges arise due to the diverse etiologies of RIF. Improving embryo implantation rates has become a major challenge in improving the clinical outcomes of IVF/ICSI-ET patients. Therefore, further research on RIF patients is necessary. The definition of RIF lacks a unified international standard. Currently, most experts accept the criteria based on the patient’s age, the number of failed IVF/ICSI cycles, and the number of good-quality embryos transferred (13). In China, the 2023 expert consensus defines RIF as the failure to achieve clinical pregnancy after transferring at least three good-quality embryos in three fresh or frozen cycles (14). The etiology of embryo implantation failure is complex, diverse, and partially unknown (15). Some studies have reported that the number of previous embryo implantation failures is an independent risk factor affecting implantation rates (5, 8, 9, 12). However, other studies have suggested that patient outcomes in IVF/ICSI cycles are not significantly related to the number of embryo transfer cycles (16). In our study, we found that patients without any implantation failures had significantly higher implantation rates, clinical pregnancy rates, and live birth rates, and a lower rate of early spontaneous abortion compared to those in the other three groups. The pregnancy outcomes were comparable between one and two previous embryo implantation failure with no significant differences in implantation rate, clinical pregnancy rate, and live birth rate (see attachment for details). However, the outcomes of patients with ≥3 previous implantation failures were worse than those of the other three groups of patients.

It is well known that age is the most critical factor affecting the development of oocytes and the quality of embryos. As female age increases, the decline in ovarian function and the increased probability of aneuploidy, she is prone to embryo developmental delay and stagnation, which can lead to embryo implantation failure, and the implantation rate decreases gradually (17, 18). We selected patients less than 40 years old and adjusted age by multiple logistic regression to avoid the impact of age on pregnancy outcomes. Impaired endometrial receptivity is also an important factor that causes embryo implantation failure (19). Therefore, patients underwent hysteroscopy after the first cycle of implantation failure to exclude interference from endometrial polyps, uterine adhesions, and chronic endometritis on implantation rates (20).

The success of ART largely depends on the quality of the embryo and the receptivity of the endometrium. In this study, the implantation rate, the clinical pregnancy rate and the live birth rate of patients underwent first embryo transfer cycle were 49.97%, 64.46% and 54.78% respectively, which was a higher rate of successful pregnancy compared to those who had previous failed cycles, which is similar to Shapiro B S study (21). Patients with one or two previous embryo implantation failures exhibited comparable pregnancy outcomes, while patients with three or more previous failures showed significantly lower rates of embryo implantation, clinical pregnancy, and live birth. Patients with three or more previous failures was RIF patients, whose infertility is often caused by multiple factors, including maternal endocrine and immune disorders and thrombophilia (22). Our cohort did not undergo etiology-related investigations and treatments for RIF patient. However, we have since put in place etiologic screening and appropriate treatment for patients with four or more embryo transfer cycles. Our findings highlight the need for further research into RIF patients, as the etiology of this condition is complex and not well understood. Therefore, patients with ≥3 previous implantation failures are recommended to continue subsequent cycles only after examination and treatment to improve pregnancy outcomes.

### Effect of the number of previous embryo implantation failures on early spontaneous abortion rate in IVF/ICSI-ET assisted conception

Spontaneous abortion is a common complication of pregnancy in obstetrics and gynecology. It is known that spontaneous abortion after IVF/ICSI-ET-assisted clinical pregnancy reduces the live birth rate. However, it is uncertain how the number of previous embryo implantation failures affects the rate of spontaneous abortion after assisted clinical pregnancy (10, 23). Previous studies have yielded conflicting results regarding the impact of the number of embryo transfer cycles on the rate of spontaneous abortion after clinical pregnancy in IVF/ICSI-assisted conception. Some studies have shown no significant change in the spontaneous abortion rate as the number of cycles increased, while others have demonstrated an increased risk of spontaneous abortion in patients with multiple embryo transfer cycles, which partially supports the results of our study (24, 25). In our study, we found that the early spontaneous abortion rate was lowest in the first embryo transfer group. Patients with ≥3 embryo implantation failures had a significantly higher early spontaneous abortion rate of 28.07%, which may be due to the fact that these patients are RIF population, and the etiology of RIF and recurrent spontaneous abortion (RSA) is similar (26). The etiology of RSA involves chromosomal or genetic abnormalities, anatomical abnormalities, autoimmune diseases, prethrombotic state (PTS), endocrine factors, infectious factors, male factors, and psychological factors (27). However, the etiological examination and correction were not carried out in our study for patients with ≥3 embryo implantation failures, which may explain the high risk of spontaneous abortion in these patients.

However, there are some limitations in this study. First, our study was a single-center, retrospective study and there might be some confounders that we did not control for. In addition, we prioritized some pregnancy outcomes and did not investigate neonatal outcomes. In the future, our findings need to be validated by expanding the sample size or by high-quality randomized clinical trial studies.

In conclusion, the number of previous embryo implantation failures is an independent factor affecting implantation rate, clinical pregnancy rate, spontaneous abortion rate and live birth rate of patients underwent IVF/ICSI-ET. Based on our study, patients with ≥3 previous implantation failures are recommended to undergo etiology-related investigations and treatments to continue subsequent cycles to improve pregnancy outcomes. Investigations for RIF includes: 1. General risk factors such as old age, poor lifestyle, smoking, etc. 2. Immune factors include autoimmunity and alloimmunity; 3. Prethrombotic states include hereditary and acquired thrombophilia; 4 Endometrial receptivity test 5. Factors of infection; 6 Reproductive anatomy; 7. Endocrine factors; 8. Male factor; 9. Chromosomes. Treatments of RIF: 1. General treatment such as weight control, healthy diet and appropriate exercise; 2.IVF-ET program optimization; 3. Regulation of immune disorders (glucocorticoids, low molecular weight heparin, immunoglobulin, etc.); 4. Low molecular weight heparin for treatment of pre-thrombotic state; 5. Doxycycline and metronidazole for chronic endometritis; 6. Treatment of submucosal myoma, polyps and hydrosalpinx and other normal anatomical structure abnormalities; 7. The man controls his weight, reduces smoking, etc. 8. Encourage both couples or those with chromosome abnormalities to perform PGT treatment.

## Data availability statement

The original contributions presented in the study are included in the article/supplementary material. Further inquiries can be directed to the corresponding author.

## Ethics statement

The studies involving human participants were reviewed and approved by Henan Medical Ethics Committee of Provincial People’s Hospital. The patients/participants provided their written informed consent to participate in this study.

## Author contributions

YF and HQ conception and design, review and final approval of the version to be published. FJ, CT, XH analyses the data. YF and HQ draft and revise the article. YF and FJ collect and analyze the data. All authors contributed to the article and approved the submitted version.
